# Long-term Response of *Helicobacter pylori* Antibody Titer After Eradication Treatment in Middle-aged Japanese: JPHC-NEXT Study

**DOI:** 10.2188/jea.JE20200618

**Published:** 2023-01-05

**Authors:** Shiori Tanaka, Atsushi Goto, Kazumasa Yamagishi, Motoki Iwasaki, Taiki Yamaji, Taichi Shimazu, Hiroyasu Iso, Isao Muraki, Nobufumi Yasuda, Isao Saito, Tadahiro Kato, Kiyoshi Aoyagi, Kazuhiko Arima, Kiyomi Sakata, Kozo Tanno, Manami Inoue, Norie Sawada, Shoichiro Tsugane

**Affiliations:** 1 Epidemiology and Prevention Group, Center for Public Health Sciences, National Cancer Center, Tokyo, Japan; 2 Department of Health Data Science, Graduate School of Data Science, Yokohama City University, Yokohama, Japan; 3 Department of Public Health Medicine, Faculty of Medicine, and Health Services Research and Development Center, University of Tsukuba, Ibaraki, Japan; 4 Ibaraki Western Medical Center, Ibaraki, Japan; 5 Public Health, Department of Social Medicine, Osaka University Graduate School of Medicine, Osaka, Japan; 6 Department of Public Health, Kochi University Medical School, Kochi, Japan; 7 Department of Public Health and Epidemiology, Faculty of Medicine, Oita University, Oita, Japan; 8 Center for Education and Educational Research, Faculty of Education, Ehime University, Ehime, Japan; 9 Department of Public Health, Nagasaki University Graduate School of Biomedical Sciences, Nagasaki, Japan; 10 Department of Hygiene and Preventive Medicine, Iwate Medical University, Iwate, Japan

**Keywords:** *Helicobacter pylori*, antibody, eradication treatment, cohort, Japan

## Abstract

**Background:**

*Helicobacter pylori* (*H. pylori*) is an established causative factor of gastric cancer. Although the expansion of insurance coverage has led to an increase in the number of patients treated for *H. pylori*, the population impact of eradication treatment for *H. pylori* has been scarcely investigated. This study aimed to clarify the long-term responses of *H. pylori* antibody titer after eradication treatment using large scale cross-sectional data from the Japan Public Health Center-based Prospective Study for the Next Generation (JPHC-NEXT Study).

**Methods:**

A total of 55,282 Japanese participants aged 40 to 74 years residing in 16 areas provided blood samples from 2011 through 2016. From these, treated (*n* = 6,276) and untreated subjects who were seropositive for *H. pylori* or had serological atrophy (*n* = 22,420) formed the study population (*n* = 28,696). Seropositivity was defined as an anti-*H. pylori* IgG titer of ≥10 U/mL. Antibody level was compared among subjects according to self-reported treatment history as untreated, and treated for less than 1 year (<1Y), 1 through 5 years (1–5Y), and 6 or more years ago (6Y+).

**Results:**

Median serum antibody titer was 34.0 U/mL, 7.9 U/mL, 4.0 U/mL, and 2.9 U/mL for the untreated, <1Y, 1–5Y, and 6Y+ groups, respectively. While those treated for *H. pylori* within the previous year had a 76.8% lower antibody titer compared to untreated subjects, approximately 41% of subjects were still seropositive.

**Conclusion:**

A significant reduction in *H. pylori* antibody titer occurs within 1 year after eradication treatment, but that a long period is needed to achieve complete negative conversion.

## INTRODUCTION

Gastric cancer is the fifth-most common cancer globally, and approximately 89% of cases are attributable to *H. pylori* infection.^1^ Japan is one of the countries with a high incidence of gastric cancer, as well as a high prevalence of *H. pylori* infection.^2^ Although improvements in public hygiene and environmental conditions have led to a significant decline in *H. pylori* infection,^3^^,^^4^ gastric cancer remains a major public health challenge in Japan.^5^^,^^6^

Previous reports have suggested that the eradication of *H. pylori* reduces the risk of gastric cancer.^7^^–^^14^ Accordingly, the Japanese government approved coverage of eradication treatment for *H. pylori* gastritis under the public health insurance in 2013, resulting in a dramatic increase in the number of patients receiving *H. pylori* eradication treatment.^15^^,^^16^ Although the population impact of eradication treatment may affect the future incidence of gastric cancer, chronological change in *H. pylori* antibody titer after eradication using large-scale population data has been scarcely investigated. Of note, antibody titer levels after eradication may be a potential predictor of post-eradication gastric cancer, with substantial impact on population health.

Here, to investigate the long-term response of *H. pylori* antibody titers following eradication, we conducted a cross-sectional analysis of baseline data from a large cohort study by comparing the history of eradication treatment in middle-aged and elderly Japanese.

## METHODS

### Study participants

The study was conducted using the baseline survey in The Japan Public Health Center-based Prospective Study for the Next Generation (JPHC-NEXT Study). The details of the study design have been described elsewhere.^17^ The purpose and human rights regarding blood collection were informed to each participant in person, and written consent was obtained. A total of 55,282 Japanese participants aged 40 to 74 years residing in 16 municipalities (2 in Iwate, 1 in Akita, 7 in Nagano, 1 in Ibaraki, 2 in Kochi, 1 in Ehime, and 2 in Nagasaki prefectures) in Japan provided blood samples between 2011 and 2016. At the same time, the participants were surveyed using a self-administered questionnaire on their history of gastric cancer, as well as receipt of *H. pylori* eradication treatment as follows: (1) no history; and (2) receipt less than 1 year; (3) 1 to 5 years ago; and (4) 6 or more years ago. The study protocol was approved by the Institutional Review Board of the National Cancer Center Japan (Approval number: 2011-186).

### Blood collection

We used sera that were derived from biospecimens obtained in the health examinations or sera especially collected for this study. Immunoglobulin (IgG) antibody titer against *H. pylori* was measured using enzyme immunoassay (EIA) (E Plate “Eiken” HP Antibody; Eiken Chemical Co., Ltd., Tokyo, Japan), while pepsinogen (PG) concentration was measured using latex agglutination (LA) (LSI Medience Co., Tokyo, Japan). This commercial kit, which is widely used to measure anti-*H. pylori* IgG titer in Japan, has a lower limit of detection of 3 U/mL. Sensitivity and specificity are approximately 91% and 97%, respectively.^18^ Seropositivity was defined as an anti-*H. pylori* IgG titer of ≥10 U/mL. Serological atrophic gastritis (AG) status was set as a combination of serum PG I level ≤70 ng/mL and PG I/II ratio ≤3.0.^19^^,^^20^

### Study population

**Figure 1.**
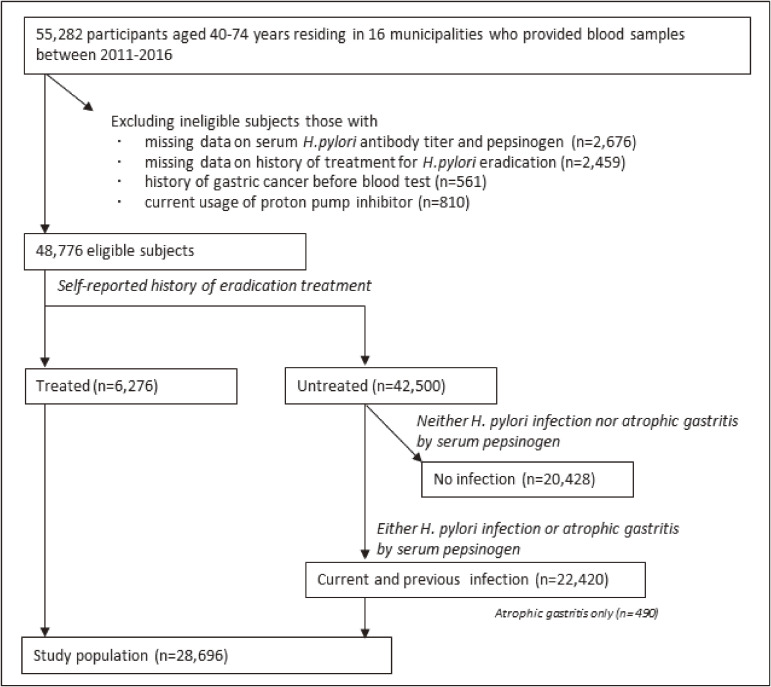
Study flow

Of 55,282 participants, those who were missing data on *H. pylori* antibody titers, serum PG, and history of eradication treatment (*n* = 5,135) or had a history of gastric cancer before blood collection (*n* = 561) were excluded (Figure 1). Because proton pump inhibitors (PPI) alter the serum PG concentration, we identified those who were taking a PPI prescribed by a medical doctor at the time of enrollment using the baseline questionnaire, resulting in the exclusion of 810 subjects. A total of 48,776 eligible subjects were classified into treated (*n* = 6,276) or untreated (*n* = 42,500) groups according to their self-report. To perform a quantitative comparison of *H. pylori* antibody titers between pre and post-treatment at the population level, we used untreated subjects with seropositivity for *H. pylori* or serological AG by pepsinogen as reference in the analysis. Of 42,500 untreated subjects, we excluded those who were not seropositive or classified with AG by pepsinogen (*n* = 20,080). As a result, treated (*n* = 6,276) and untreated (*n* = 22,420; AG by pepsinogen only: *n* = 490) subjects who were previously infected with *H. pylori* formed the study population (*n* = 28,696).

### Statistical analysis

Antibody titers against and seroprevalence of *H. pylori* in the study participants were assessed based on treatment history as (1) none (untreated group); and (2) treatment less than 1 year (<1Y group); (3) 1 to 5 years ago (1–5Y group); and (4) 6 or more years ago (6Y+ group). *H. pylori* antibody titers were classified into three groups of <3 U/mL, ≥3 U/mL to <10 U/mL, and ≥10 mL, as low-negative, high-negative, and positive, respectively.^21^ Continuous variables were expressed as the mean (standard deviation [SD]) or median (interquartile range [IQR]) when distributions were skewed. Medians were compared using unadjusted quantile regression by assigning categories as an ordinal variable. The figures for quartiles of antibody titer and seroprevalence were presented using cubic spline interpolation. The antibody distributions were further assessed by age at blood test and sex. Because a previous study has reported that the prevalence of previous infection-induced AG was around 10.0–19.8% in Japan irrespective of negative conversion,^22^ we further excluded subjects showing a serum titer of 3 to <10 U/mL to examine the influence of unintentional eradication among the untreated group. A *P*-value of <0.05 was considered statistically significant. We used STATA version 16 for all analyses (StataCorp LLC, College Station, TX, USA).

## RESULTS

Among 28,696 subjects (mean age, 61.7 years; 44.8% male), a total of 21.9% were treated for *H. pylori* (Table 1). Mean age did not differ among groups. Men were more commonly treated than women (29.5% vs 22.4%). Compared to the untreated group, PG I and PG II were lower in all treated groups. With years since treatment, the PG I/II ratio increased while the prevalence of AG by serum pepsinogen decreased.

**Table 1.** Basic characteristics according to the self-reported treatment history for *H. pylori* among ever-infected subjects in the JPHC-NEXT Study

	Overall	Untreated^a^	Years from treatment^a^		

			<1Y	1–5Y	6Y+
Total, *n* (%)	28,696 (100.0)	22,420 (78.1)	1,100 (3.8)	2,889 (10.1)	2,287 (8.0)
Age, years, mean (SD)	61.7 (8.4)	61.7 (8.5)	61.7 (8.3)	61.6 (8.1)	61.5 (7.8)
Sex					
Male, *n* (%)	12,857 (100.0)	9,583 (74.5)	484 (3.8)	1,429 (11.1)	1,361 (10.6)
Female, *n* (%)	15,839 (100.0)	12,837 (81.0)	616 (3.9)	1,460 (9.2)	926 (5.8)
Serum pepsinogen					
PG I, IQR, ng/mL	60.2 (36.5–74.6)	61.4 (36.8–77.4)	43.0 (31.2–58.8)	45.7 (34.4–60.7)	52.3 (41.0–69.7)
PG II, IQR, ng/mL	19.4 (9.9–25.4)	21.7 (12.7–27.8)	8.2 (6.4–11.3)	8.5 (6.7–11.5)	9.8 (7.6–13.0)
PG I/II ratio, mean (SD)	3.6 (2.0)	3.1 (1.7)	5.1 (1.9)	5.4 (1.8)	5.5 (1.7)
Serum AG^b^, *n* (%)^c^	10,001 (34.9)	9,565 (42.7)	133 (12.1)	181 (6.3)	122 (5.3)

AG, atrophic gastritis; IQR, interquartile range; *n*, number; PG, pepsinogen; SD, standard deviation.

^a^Subjects were classified according to their self-reported treatment history for *H. pylori* as follows: none: untreated; treated less than 1 year: <1Y; treated 1 to 5 years ago: 1–5Y; and treated 6 or more years ago: 6Y+.

^b^Defined as a combination of the serum PG I level ≤70 ng/mL and PG I/II ratio ≤3.0.

^c^Percentage of atrophic gastritis by pepsinogen.

**Figure 2.**
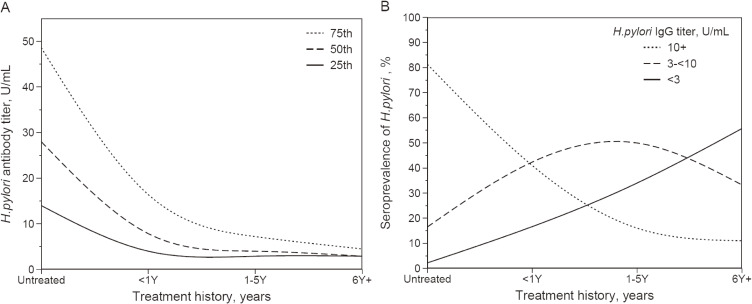
Quartiles of *H. pylori* antibody titer and seroprevalence according to history of eradication treatment for *H. pylori* among ever-infected subjects in the JPHC–NEXT Study. Subjects were classified according to self-reported treatment history for *H. pylori* as follows: none: untreated; treated less than 1 year: <1Y; treated 1 to 5 years ago: 1–5Y; and treated 6 or more years ago: 6Y+. A) Spline-smoothed lines connecting the 25^th^, 50^th^, and 75^th^ percentiles of antibody titers in each group, respectively. B) Spline-smoothed lines for seroprevalence of *H. pylori* according to treatment history. *H. pylori* antibody titers of <3 U/mL, ≥3 to <10 U/mL, and ≥10 U/mL were defined as low-negative, high-negative and positive, respectively.

Table 2 presents antibody titers according to treatment history. Median serum antibody titer level was 34.0 (IQR, 14.0–48.6) U/mL, 7.9 (IQR, 4.0–16.4) U/mL, 4.0 (IQR, 3.0–7.2) U/mL, and 2.9 (IQR, 2.9–4.5) U/mL for the untreated, <1Y, 1–5Y, and 6Y+ groups, respectively. Median *H. pylori* antibody titer was significantly decreased overtime since treatment in all subjects and both sexes (*P* < 0.01). Compared to the untreated group, median antibody titer of the <1Y, 1–5Y, and 6Y+ groups was decreased by 76.8%, 88.2%, and 91.5%, respectively. Percentages of low-negative subjects in the <1Y, 1–5Y, and 6Y+ groups were 16.6%, 34.0% and 55.7%, respectively. High-negative subjects accounted for 42.4%, 50.0% and 33.3% of the <1Y, 1–5Y, and 6Y+ groups. Meanwhile, those who were seropositive were 41.0%, 16.0%, and 11.0% in the <1Y, 1–5Y, and 6Y+ groups. Those who were both *H. pylori*-negative and classified with AG by serum pepsinogen were 1.8%, 1.0%, and 1.0% in the <1Y, 1–5Y, and 6Y+ groups, respectively. Figure 2A shows quartiles of antibody titer by group. The slope of the decline in titers between the 75^th^ vs 25^th^ percentile subjects was quite different, suggesting that pre-treatment antibody titer may have influenced the quantitative response to treatment. The smoothed-spline curve of seroprevalence for *H. pylori* is also shown in Figure 2B. The prevalence of seronegativity continued to increase over time from treatment, while the ratio of subject with antibody titers of 3 to <10 U/mL remained high among the <1Y and 1–5Y groups.

**Table 2.** Anti-*H. pylori* IgG titers and seroprevalence according to self-reported treatment history for *H. pylori* among ever-infected subjects in the JPHC-NEXT Study

	Untreated^a^	Years from treatment^a^			*P*-value^b^
	
		<1Y	1–5Y	6Y+	
All	*n* = 22,420	*n* = 1,100	*n* = 2,889	*n* = 2,287	
*H. pylori* antibody titer, IQR, U/mL	34.0 (14.0–48.6)	7.9 (4.0–16.4)	4.0 (3.0–7.2)	2.9 (2.9–4.5)	<0.01
<3 U/mL^c^, *n* (%)	490 (2.2)	183 (16.6)	982 (34.0)	1,274 (55.7)	<0.01
3–<10 U/mL^c^, *n* (%)	3,700 (16.5)	466 (42.4)	1,444 (50.0)	761 (33.3)	
≥10 U/mL^c^, *n* (%)	18,230 (81.3)	451 (41.0)	463 (16.0)	252 (11.0)	

Men	*n* = 9,583	*n* = 484	*n* = 1,429	*n* = 1,361	
*H. pylori* antibody titer, IQR, U/mL	32.8 (13.7–45.0)	7.6 (4.3–14.3)	3.9 (3.0–6.8)	2.9 (2.9–4.5)	<0.01
<3 U/mL^c^, *n* (%)	197 (2.1)	80 (16.5)	502 (35.1)	757 (55.6)	<0.01
3–<10 U/mL^c^, *n* (%)	1,562 (18.4)	221 (45.7)	721 (50.5)	454 (33.4)	
≥10 U/mL^c^, *n* (%)	7,824 (81.6)	183 (37.8)	206 (14.4)	150 (11.0)	

Women	*n* = 12,837	*n* = 616	*n* = 1,460	*n* = 926	
*H. pylori* antibody titer, IQR, U/mL	36.0 (14.3–51.1)	8.0 (3.9–18.5)	4.2 (3.0–7.6)	2.9 (2.9–4.5)	<0.01
<3 U/mL^c^, *n* (%)	293 (2.3)	103 (16.7)	480 (32.9)	517 (55.8)	<0.01
3–<10 U/mL^c^, *n* (%)	2,138 (16.7)	245 (39.8)	723 (49.5)	307 (33.2)	
≥10 U/mL^c^, *n* (%)	10,406 (81.1)	268 (43.5)	257 (17.6)	102 (11.0)	

IQR, interquartile range; *n*, number.

^a^Subjects were classified according to their self-reported treatment history for *H. pylori* as follows: none: untreated; treated less than 1 year ago: <1Y; treated 1 to 5 years ago: 1–5Y; and treated 6 or more years ago: 6Y+.

^b^Chi-square test for categorical variables and unadjusted quantile regression to compare median values.

^c^*H. pylori* antibody titer of <3 U/mL, ≥3 to <10 U/mL, and ≥10 U/mL were defined as low-negative, high-negative and positive, respectively.

Median antibody titer was slightly decreased with age among the untreated group (*P* = 0.02), while little difference was seen among age groups after treatment, irrespective of years since treatment (eTable 1). Seroprevalence did not much differ among age groups (eTable 2). Median antibody titers and seroprevalence tended not to differ by sex.

Sensitivity analysis, in which untreated subjects with serum titers 3 to <10 U/mL were excluded, showed that the median antibody titer of the untreated group was 34.3 (IQR, 21.9–54.6) U/mL and that the percentage decrease in antibody titer between the untreated and <1Y groups was 77.0%.

## DISCUSSION

In this study, we assessed the serological response of anti-*H. pylori* IgG titer after eradication treatment using large-scale cross-sectional data from a cohort study. The median antibody titer was lower in the group with the longest period since eradication treatment. A previous study with successful eradication cases using the same kit reported a similar result,^23^ indicating that any bias arising from the unclear status of *H. pylori* elimination in this study did not significantly affect our results.

Compared with the untreated group, median antibody titer was decreased by 76.8% in the <1Y, 88.2% in the 1–5Y, and 91.5% in the 6Y+ groups. This indicated that a significant reduction in antibody titer occurred within 1 year of treatment and then continued to decline over time. Previous studies reported that IgG level decreased from 2 months to 1 year after successful treatment by approximately 16–87%.^24^^–^^26^ Although this initial drop following treatment was reported to occur irrespective of eradication results, the degree of the initial drop and the following serological course depended on whether the eradication had been successful or not.^27^ Those with successful eradication continued to have low IgG titers due to the disappearance of *H. pylori* from the gastric mucosa; conversely, titers were slightly elevated among those in whom eradication failed.^25^^–^^27^

In this study, 59.0% of subjects in the <1Y group were found to be seronegative. Previous study reported negative conversion rates of 35–62% at 1 year from *H. pylori* elimination.^25^^,^^26^^,^^28^^,^^29^ It took 17.9 months for those with an intermediate level of antibody titer before eradication to convert to negative, and 42.8 months for those with a higher level.^30^ Complete negative conversion was usually not observed within 1 year after eradication.^31^^,^^32^

Approximately 84% and 89% of the 1–5Y and 6Y+ groups, respectively, with IgG titers became seronegative. Previous studies focused on Japanese patients showed varied results on the seronegative rate. Shirai reported that 62% of patients had an antibody titer level below the cut-off within 2 years.^33^ Only 7.5% of patients in the highest antibody titer group before elimination was seronegative 3 years after eradication, versus 60% in the lowest titer group.^34^ Ohara reported that no seropositive cases were seen from 4 years after successful eradication,^23^ but that no seropositive cases during a long follow-up is unlikely because of the possibility of reinfection and recurrence. Matsuhisa demonstrated a cumulative seronegative rate of only 23.5% during the 6 years since *H. pylori* elimination.^30^ The different results shown in studies might have been due to differences in the usage of kits, different conditions before elimination in patients, and loss to follow-up due to the disappearance of symptoms.^30^

We showed that 42.4%, 50.0%, and 33.3% of subjects in the <1Y, 1–5Y, and 6Y+ groups, respectively, had a high-negative antibody titer level. Similar results were reported in a study that focused on subjects with successful eradication; high-negative cases accounted for 50–65% between 1–9 years after successful eradication.^23^ Approximately 41.0%, 16.0%, and 11.0% for the <1Y, 1–5Y, and 6Y+ groups, respectively, were seropositive in this study. A quantitative association between pre-eradication *H. pylori* antibody titer and time to seronegative conversion was reported,^31^ indicating that the high-negative and seropositive subjects may have had a higher antibody titer before treatment. We observed a difference in the slope of reduction between subjects in the 25^th^ and 75^th^ percentiles of antibody titers. A slow decrease in antibodies might also reflect delayed eradication due to invasion of the bacteria in the mucosa and translocation to the gastric lymph nodes.^35^ Another reason for incomplete negative conversion might include eradication failure, recurrence, and reinfection.^36^ As the success rate in first-line eradication treatment is 75–90%,^37^^–^^41^ most seropositivity among the 6Y+ group may be explained by treatment failure. Although new infection in adulthood and recurrence is not common,^42^^–^^44^ a return to seropositivity in subjects with successful eradication is possible.^36^ A study with long follow-up revealed that recurrence could occur within 4 years after *H. pylori* eradication and that the annual reinfection rate was 0.2–2.0% in Japanese,^42^^,^^43^ albeit that reinfection and recurrence were not completely distinguished.

Although previous studies indicate that the success rate of eradication varies by gender and age because of antimicrobial susceptibility and differences in compliance,^45^^,^^46^ we observed that median antibody titer and seroprevalence among those treated for *H. pylori* did not much differ by gender or age.

Given that negative conversion of antibody takes more than 1 year after successful eradication,^47^ a guideline indicates that measurement of IgG titer is not a suitable way of determining prompt eradication results.^48^ Used alone, antibody measurement may lead to a misdiagnosis of failed primary eradication, resulting in unnecessary second treatment.^49^ Quantitative comparison of antibody titers pre- and post-eradication may nevertheless serve as a monitor in evaluating the short- and long-term impact with regard to eradication, recurrence, reinfection, and disease prediction.^27^^,^^36^ Elevated antibodies after successful eradication may be associated with persistent mild chronic inflammation.^50^ Moreover, a higher level of IgG after successful eradication was observed in a gastric cancer group compared to a no-gastric cancer group.^51^ Once the association between post-eradication antibody titers for *H. pylori* and risk of gastric cancer is clarified, measurement of antibody titers after eradication will have a major impact on population health.

### Strengths and limitations

This study is the largest report on the long-term response of *H. pylori* antibody titers after eradication treatment in middle-aged and elderly Japanese. However, several methodological limitations warrant mention. First, the history of eradication treatment was based on self-report, which might have introduced misclassification due to misunderstanding therapy with other medications. We addressed this issue by confirming the history of eradication treatment using the follow-up questionnaire and medical claims data in the Saku area (*n* = 2,443). The sensitivity, specificity, and positive predictive values of a self-reported eradication history were 96.7%, 89.1%, and 78.8%, respectively, showing the validity of the usage of a self-reported history of eradication treatment for *H. pylori*. Besides, the higher PG I/II ratio seen in the treated groups compared to the untreated group may support *H. pylori* eradication.^48^^,^^52^ Second, we were unable to verify whether eradication treatment had been successful or not. The possible inclusion of subjects with failed eradication might have overestimated the seropositive rate and antibody titer level because the antibody titer of patients with unsuccessful eradication should have been higher than that of those with successful eradication.^36^ Third, unexpected eradication resulting from unintended eradication and spontaneous disappearance of *H. pylori* as a result of advanced AG cannot be excluded completely, especially among the untreated group.^53^^,^^54^ Additionally, it is important to note that there is no definitive method to identify unintended elimination of *H. pylori* among those who do not have a history of eradication even using endoscopy, serology, or a medical examination.^22^ Fourth, we were unable to identify which *H. pylori* eradication regimens the subjects received. Antibody response may have varied among procedures. Fifth, quantitative comparison before and after treatment was not derived from serial blood samples from individuals. Last, the study subjects were recruited mainly from annual health checkup surveys provided by the local government for general community residents, which might have introduced selection bias.

In summary, this study demonstrated the serological responses of eradication treatment for *H. pylori* at the population level. While the subjects treated with *H. pylori* in the previous year had a 76.8% lower antibody titer than untreated cases, approximately 40% were still seropositive. An initial drop in antibody titer occurs within 1 year after treatment, but a long period is required to achieve negative conversion. As there were several methodological limitations, our study provides insights for further investigation of the use of chronological change in *H. pylori* antibody titer as a potential marker of gastric cancer risk.

## SUPPLEMENTARY MATERIAL

eTable 1. Median (interquartile) of *H. pylori* antibody titers according to the self-reported treatment history for *H. pylori* by age at blood test among ever-infected subjects in the JPHC-NEXT Study

eTable 2. Seroprevalence of *H. pylori* according to the self-reported treatment history for *H. pylori* by age at blood test among ever-infected subjects in the JPHC-NEXT Study

eFigure 1. Quartiles of *H. pylori* antibody titers according to the self-reported treatment history for *H. pylori* by age at blood test among ever-infected subjects in the JPHC–NEXT.

eFigure 2. The seroprevalence of *H. pylori* according to the self-reported treatment history for *H. pylori* by age at blood test among ever-infected subjects in the JPHC–NEXT Study
