# Supplementary material for: Long-term Response of Helicobacter pylori Antibody Titer After Eradication Treatment in Middle-aged Japanese: JPHC-NEXT Study
Source: J Epidemiol. 2023 Jan 5;33(1):1–7. doi: 10.2188/jea.JE20200618 (PMC9727212; doi:10.2188/jea.JE20200618)
Supplement: Supplementary file 1 [file je-33-001-s001.pdf]

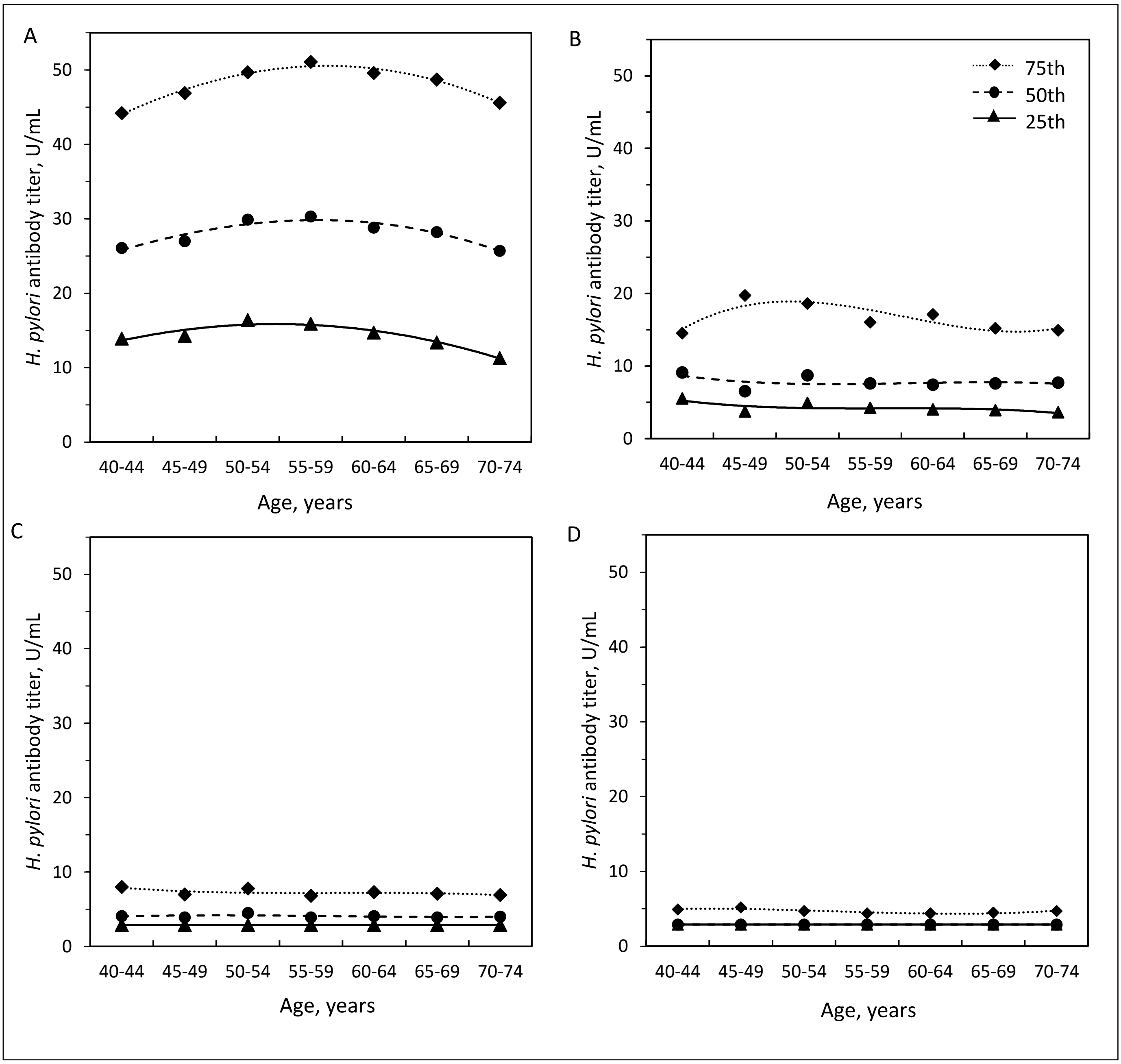

eFigure 1. Quartiles of *H. pylori* antibody titers according to the self-reported treatment history for *H. pylori* by age at blood test among ever-infected subjects in the JPHC –NEXT.

Spline-smoothed lines connecting the 25th, 50th, and 75th percentiles of antibody titer in each birth cohort, respectively. A (Untreated): those who reported no treatment history for *H. pylori* with seropositive or serological atrophic gastritis; B(<1Y): those who reported receiving treatment within 1 year; C (1–5Y): those who reported receiving treatment 1–5 years ago among ever infected subjects; D (6Y+): those who reported receiving treatment 6 or more years ago.

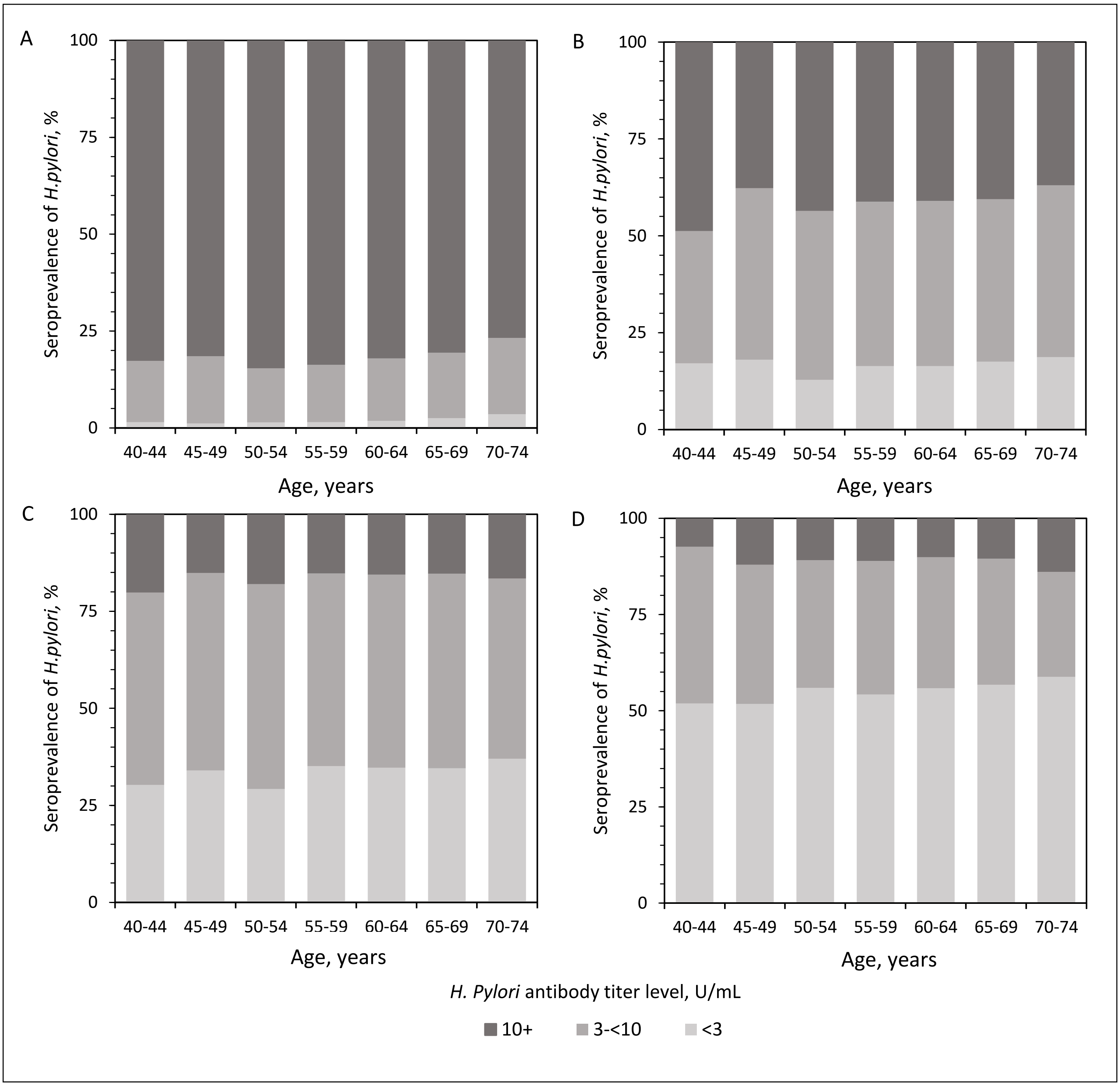

eFigure 2. The seroprevalence of *H. pylori* according to the self-reported treatment history for *H. pylori* by age at blood test among ever-infected subjects in the JPHC –NEXT Study

A (Untreated): those who reported no treatment history for *H. pylori* with seropositive or serological atrophic gastritis; B(<1Y): those who reported receiving treatment within 1 year; C (1–5Y): those who reported receiving treatment 1–5 years ago among ever infected subjects; D (6Y+): those who reported receiving treatment 6 or more years ago.

eTable 1. Median (interquartile) of *H. pylori* antibody titers according to the self-reported treatment history for *H. pylori* by age at blood test among ever-infected subjects in the JPHC-NEXT Study

Treatment history[a]	Age at blood test, years							Total	*P*-value[b]
	40–44	45–49	50–54	55–59	60–64	65–69	70–74		
All subjects									
Untreated	16.2 (14.1 – 44.2)	27.0 (14.4 – 47.0)	29.9 (16.4 – 49.7)	30.4 (16.1 – 51.3)	28.9 (14.8 – 49.8)	28.3 (13.4 – 48.8)	25.7 (11.3 – 45.6)	28.0 (14.0 – 48.6)	0.02
<1Y	8.4 (4.7 – 14.2)	6.7 (3.6 – 20.2)	8.6 (4.8 – 17.9)	7.6 (4.1 – 15.9)	7.4 (3.9 – 17.1)	7.7 (3.9 – 15.2)	8.3 (3.8 – 17.3)	7.9 (4.0 – 16.4)	0.80
1–5Y	4.3 (2.9 – 8.4)	3.9 (2.9 – 7.0)	4.5 (2.9 – 7.8)	3.9 (2.9 – 6.8)	4.1 (2.9 – 7.2)	4.0 (2.9 – 7.1)	4.0 (2.9 – 6.9)	4.0 (2.9 – 7.2)	0.56
6Y+	2.9 (2.9 – 4.9)	2.9 (2.9 – 5.2)	2.9 (2.9 – 4.7)	2.9 (2.9 – 4.4)	2.9 (2.9 – 4.3)	2.9 (2.9 – 4.7)	2.9 (2.9 – 4.8)	2.9 (2.9 – 4.5)	1.00
Men									
Untreated	27.0 (14.9 – 46.9)	27.1 (14.2 – 45.7)	27.1 (16.5 – 46.3)	27.3 (15.8 – 45.2)	26.7 (14.1 – 46.4)	26.1 (12.8 – 44.5)	24.4 (11.1 – 42.5)	26.2 (13.7 – 45.0)	<0.01
<1Y	8.8 (5.5 – 13.0)	6.7 (3.5 – 19.2)	7.5 (4.7 – 16.0)	7.0 (3.6 – 15.6)	7.4 (4.1 – 13.1)	7.5 (4.5 – 12.8)	8.6 (4.3 – 17.3)	7.6 (4.3 – 14.3)	0.80
1–5Y	4.2 (2.9 – 8.4)	3.7 (2.9 – 5.1)	4.0 (2.9 – 7.5)	3.7 (2.9 – 5.7)	4.1 (2.9 – 7.4)	3.7 (2.9 – 6.3)	3.9 (2.9 – 6.9)	3.9 (2.9 – 6.8)	1.00
6Y+	3.0 (2.9 – 5.0)	2.9 (2.9 – 4.5)	2.9 (2.9 – 4.2)	2.9 (2.9 – 4.5)	2.9 (2.9 – 4.4)	2.9 (2.9 – 4.5)	2.9 (2.9 – 5.5)	2.9 (2.9 – 4.5)	1.00
Women									
Untreated	25.9 (13.2 – 41.4)	27.0 (14.9 – 48.2)	31.8 (16.4 – 51.2)	32.0 (16.3 – 53.9)	30.4 (15.4 – 53.3)	30.2 (14.0 – 51.6)	27.0 (11.4 – 48.8)	29.7 (14.3 – 51.1)	0.92
<1Y	8.0 (4.6 – 17.1)	6.4 (3.6 – 20.3)	9.3 (5.7 – 19.5)	8.4 (4.6 – 16.7)	7.4 (3.5 – 19.9)	8.0 (3.8 – 18.6)	7.9 (3.6 – 17.6)	8.0 (3.9 – 18.5)	0.91
1–5Y	4.7 (2.9 – 8.5)	4.3 (2.9 – 8.4)	5.0 (3.1 – 9.2)	4.0 (2.9 – 7.6)	4.0 (2.9 – 7.0)	4.2 (2.9 – 7.9)	4.1 (2.9 – 7.2)	4.2 (2.9 – 7.6)	0.16
6Y+	2.9 (2.9 – 4.8)	3.0 (2.9 – 5.7)	2.9 (2.9 – 5.6)	2.9 (2.9 – 4.4)	2.9 (2.9 – 4.3)	2.9 (2.9 – 4.7)	2.9 (2.9 – 4.2)	2.9 (2.9 – 4.5)	1.00

[a] Subjects were classified according to their self-reported treatment history for *H. pylori* as follows; none: Untreated; less than 1 year: <1Y; 1 to 5 years ago: 1–5Y; and 6 or more years ago: 6Y+.

[b] Unadjusted quantile regression to compare median values.

eTable 2. Seroprevalence of *H. pylori* according to the self-reported treatment history for *H. pylori* by age at blood test among ever-infected subjects in the JPHC-NEXT Study

Treatment history[a]	*H. pylori* antibody titer (U/mL)[b]	Age at blood test, years							Total	*P*-value[c]
		40–44	45–49	50–54	55–59	60–64	65–69	70–74		
All subjects										
Untreated	<3, n %	15 (1.4)	16 (1.2)	30 (1.4)	47 (1.5)	95 (1.8)	129 (2.5)	158 (3.6)	490 (2.2)	<0.01
	3－<10, n %	164 (15.5)	233 (17.1)	289 (13.8)	455 (14.5)	844 (16.0)	866 (16.8)	849 (19.6)	3,700 (16.5)	
	≥10, n %	882 (83.1)	1,114 (81.7)	1,769 (84.7)	2,626 (84.0)	4,352 (82.3)	4,162 (80.7)	3,325 (76.8)	18,230 (81.3)	
	Total, n	1,061	1,363	2,088	3,128	5,291	5,157	4,332	22,420	
<1Y	<3, n %	7 (15.6)	11 (18.3)	13 (13.0)	27 (16.5)	42 (16.9)	48 (17.1)	35 (17.3)	183 (16.6)	1.00
	3－<10, n %	18 (40.0)	26 (43.3)	44 (44.0)	70 (42.7)	103 (41.5)	118 (42.0)	87 (43.1)	466 (42.4)	
	≥10, n %	20 (44.4)	23 (38.3)	43 (43.0)	67 (40.9)	103 (41.5)	115 (40.9)	80 (39.6)	451 (41.0)	
	Total, n	45	60	100	164	248	281	202	1,100	
1–5Y	<3, n %	38 (29.7)	53 (34.2)	76 (28.8)	153 (34.9)	252 (34.8)	222 (33.8)	188 (36.0)	982 (34.0)	0.68
	3－<10, n %	63 (49.2)	80 (51.6)	140 (53.0)	218 (49.8)	363 (50.1)	334 (50.8)	246 (47.1)	1,444 (50.0)	
	≥10, n %	27 (21.1)	22 (14.2)	48 (18.2)	67 (15.3)	110 (15.2)	101 (15.4)	88 (16.9)	463 (16.0)	
	Total, n	128	155	264	438	725	657	522	2,889	
6Y+	<3, n %	43 (51.8)	60 (51.7)	116 (56.0)	203 (54.7)	345 (56.2)	303 (55.4)	204 (58.5)	1,274 (55.7)	0.65
	3－<10, n %	34 (41.0)	42 (36.2)	68 (32.9)	128 (34.5)	207 (33.7)	184 (33.6)	98 (28.1)	761 (33.3)	
	≥10, n %	6 (7.2)	14 (12.1)	23 (11.1)	40 (10.8)	62 (10.1)	60 (11.0)	47 (13.5)	252 (11.0)	
	Total, n	83	116	207	371	614	547	349	2,287	
Men										
Untreated	<3, n %	5 (1.1)	3 (0.5)	12 (1.5)	17 (1.4)	33 (1.5)	54 (2.3)	73 (3.7)	197 (2.1)	<0.01
	3－<10, n %	54 (11.8)	95 (17.0)	97 (11.8)	167 (13.5)	362 (16.4)	399 (17.3)	388 (19.5)	1,562 (16.3)	
	≥10, n %	400 (87.1)	460 (82.4)	716 (86.8)	1,050 (85.1)	1,818 (82.2)	1,855 (80.4)	1,525 (76.8)	7,824 (81.6)	
	Total, n	459	558	825	1,234	2,213	2,308	1,986	9,583	
<1Y	<3, n %	4 (18.2)	6 (21.4)	6 (13.3)	14 (21.2)	18 (15.0)	18 (15.5)	14 (16.1)	80 (16.5)	0.97
	3－<10, n %	8 (36.4)	10 (35.7)	22 (48.9)	27 (40.9)	59 (49.2)	56 (48.3)	39 (44.8)	221 (45.7)	
	≥10, n %	10 (45.5)	12 (42.9)	17 (37.8)	25 (37.9)	43 (35.8)	42 (36.2)	34 (39.1)	183 (37.8)	
	Total, n	22	28	45	66	120	116	87	484	
1–5Y	<3, n %	22 (33.8)	25 (33.8)	44 (35.2)	76 (36.7)	127 (34.4)	117 (35.1)	91 (35.5)	502 (35.1)	0.83
	3－<10, n %	29 (44.6)	41 (55.4)	62 (49.6)	103 (49.8)	185 (50.1)	177 (53.2)	124 (48.4)	721 (50.5)	
	≥10, n %	14 (21.5)	8 (10.8)	19 (15.2)	28 (13.5)	57 (15.4)	39 (11.7)	41 (16.0)	206 (14.4)	
	Total, n	65	74	125	207	369	333	256	1,429	
6Y+	<3, n %	24 (50.0)	38 (54.3)	70 (58.8)	115 (54.5)	203 (56.4)	194 (55.4)	113 (55.7)	757 (55.6)	0.46
	3－<10, n %	21 (43.8)	23 (32.9)	39 (32.8)	67 (31.8)	118 (32.8)	126 (36.0)	60 (29.6)	454 (33.4)	
	≥10, n %	3 (6.3)	9 (12.9)	10 (8.4)	29 (13.7)	39 (10.8)	30 (8.6)	30 (14.8)	150 (11.0)	

	Total, n	48	70	119	211	360	350	203	1,361	
Women										
Untreated	<3, n %	10 (1.7)	13 (1.6)	18 (1.4)	30 (1.6)	62 (2.0)	75 (2.6)	85 (3.6)	293 (2.3)	<0.01
	3－<10, n %	110 (18.3)	138 (17.1)	192 (15.2)	288 (15.2)	482 (15.7)	467 (16.4)	461 (19.7)	2,138 (16.7)	
	≥10, n %	482 (80.1)	654 (81.2)	1,053 (83.4)	1,576 (83.2)	2,534 (82.3)	2,307 (81.0)	1,800 (76.7)	10,406 (81.1)	
	Total, n	602	805	1,263	1,894	3,078	2,849	2,346	12,837	
<1Y	<3, n %	3 (13.0)	5 (15.6)	7 (12.7)	13 (13.3)	24 (18.8)	30 (18.2)	21 (18.3)	103 (16.7)	0.91
	3－<10, n %	10 (43.5)	16 (50.0)	22 (40.0)	43 (43.9)	44 (34.4)	62 (37.6)	48 (41.7)	245 (39.8)	
	≥10, n %	10 (43.5)	11 (34.4)	26 (47.3)	42 (42.9)	60 (46.9)	73 (44.2)	46 (40.0)	268 (43.5)	
	Total, n	23	32	55	98	128	165	115	616	
1─5Y	<3, n %	16 (25.4)	28 (34.6)	32 (23.0)	77 (33.3)	125 (35.1)	105 (32.4)	97 (36.5)	480 (32.9)	0.42
	3－<10, n %	34 (54.0)	39 (48.1)	78 (56.1)	115 (49.8)	178 (50.0)	157 (48.5)	122 (45.9)	723 (49.5)	
	≥10, n %	13 (20.6)	14 (17.3)	29 (20.9)	39 (16.9)	53 (14.9)	62 (19.1)	47 (17.7)	257 (17.6)	
	Total, n	63	81	139	231	356	324	266	1460	
6Y+	<3, n %	19 (54.3)	22 (47.8)	46 (52.3)	88 (55.0)	142 (55.9)	109 (55.3)	91 (62.3)	517 (55.8)	0.22
	3－<10, n %	13 (37.1)	19 (41.3)	29 (33.0)	61 (38.1)	89 (35.0)	58 (29.4)	38 (26.0)	307 (33.2)	
	≥10, n %	3 (8.6)	5 (10.9)	13 (14.8)	11 (6.9)	23 (9.1)	30 (15.2)	17 (11.6)	102 (11.0)	
	Total, n	35	46	88	160	254	197	146	926	

n, number.

[a] Subjects were classified according to their self-reported treatment history for *H. pylori* as follows; none: Untreated; less than 1 year: <1Y; 1 to 5 years ago:1─5Y; and 6 or more years ago: 6Y+.

[b] *H. pylori* antibody titer of <3 U/mL, ≥3 to <10 U/mL, and ≥10 U/mL were defined as low-negative, high-negative and positive, respectively.

[c] Chi-square tests.
